# Phenotypic Diversity in Pediatric Congenital Myasthenic Syndrome: Insights from CHRNE and DPAGT1 Variants

**DOI:** 10.3390/neurolint18060102

**Published:** 2026-05-25

**Authors:** Aya Ewida, Dima Al-Qaimari, Ubaid Shah, Nikil Sudarsan

**Affiliations:** 1 College of Medicine, University of Sharjah, Sharjah 27272, United Arab Emirates; aya.m.ewida@gmail.com (A.E.); dima.galqaimari@gmail.com (D.A.-Q.); 2 Department of Pediatric Neurology, King’s College Hospital London, Dubai 2623, United Arab Emirates; nikil.sudarsan@kch.ae; 3 College of Medicine, Gulf Medical University, Ajman 4184, United Arab Emirates

**Keywords:** congenital myasthenic syndrome, fast-channel syndrome, glycosylation defects, CHRNE gene, DPAGT1 gene, case report

## Abstract

**Introduction:** Congenital myasthenic syndrome (CMS) is a rare hereditary disorder of the neuromuscular junction caused by pathogenic variants that affect acetylcholine transmission. We report three pediatric cases with CMS, including a rare homozygous CHRNE mutation previously described only once, a novel CHRNE compound heterozygous variant, and two novel DPAGT1 variants associated with limb-girdle CMS (LG-CMS), thereby expanding the known genetic and phenotypic spectrum of the disorder. **Case presentation:** The first patient, a 4-year-old girl born to consanguineous parents, presented with bilateral ptosis and fatigable weakness since infancy. Whole-genome sequencing revealed a homozygous CHRNE variant, c.991C>T. The second patient, a 4-year-old boy born to non-consanguineous parents, presented with congenital bilateral ptosis and ophthalmoplegia without generalized weakness. Genetic analysis identified compound heterozygous CHRNE variants, c.905C>G and c.1040T>C. Both patients demonstrated marked improvement with pyridostigmine therapy. The third patient, a 3-year-old girl born to non-consanguineous parents, presented with severe limb weakness requiring assistance in walking and performing daily activities with minimal ocular involvement, suggesting a diagnosis of LG-CMS. Genetic testing identified two novel variants in the DPAGT1 gene in the compound heterozygous form, c.710G>T and c.858C>A. The initial response to pyridostigmine diminished over time. **Conclusions:** These cases underscore the phenotypic heterogeneity of CMS, even within the same genetic subtype, and expand the existing mutational spectrum of CHRNE and DPAGT1 genes. This study also highlights the essential role of molecular diagnosis in distinguishing CMS from other neuromuscular disorders. Early genetic confirmation facilitates genotype-targeted therapy, prevents inappropriate immunosuppression, and enables informed reproductive counseling.

## 1. Introduction

Congenital myasthenic syndrome (CMS) is a rare inherited disorder of the neuromuscular junction (NMJ) that leads to fatigable muscle weakness due to impaired transmission of nerve impulses to the skeletal muscles. The estimated global prevalence is approximately 9–10 per million individuals, but the true frequency is likely higher due to limited awareness and access to genetic testing and clinical overlap with congenital myopathies and ocular movement disorders. It results from pathogenic genetic variants that affect key proteins responsible for acetylcholine synthesis, release, or receptor activation [1]. CMS is a non-autoimmune condition that does not respond to immunosuppressive or antibody-directed therapies [2].

The condition can present at any age, from neonatal to adulthood, but symptoms most often appear in infancy or early childhood. Depending on the specific gene mutation, clinical signs may appear later in infancy or childhood, which can delay the diagnosis [3]. The diagnosis of CMS relies on a combination of clinical evaluations, neurophysiological testing, and genetic confirmation. It is typically performed when patients show compatible symptoms with negative anti-AChR antibodies, along with at least one of the following: electromyographic (EMG) evidence of impaired neuromuscular transmission, a positive response to pyridostigmine, or molecular genetic confirmation [3]. EMG often reveals a decremental response to repetitive nerve stimulation, while AChR and muscle-specific kinase (MuSK) antibodies remain negative, distinguishing CMS from autoimmune myasthenia gravis.

CMS represents a potentially treatable group of neuromuscular disorders in contrast to many congenital myopathies. However, treatment must be genotype-specific; therefore, accurate molecular diagnosis is crucial for effective management and appropriate genetic counseling [4]. Because inappropriate therapy may exacerbate symptoms, genetic testing plays a vital role in confirming the diagnosis and guiding appropriate treatment [2]. Advances in next-generation sequencing technologies, including whole-genome sequencing (WGS) and targeted gene panels, have improved the speed and accuracy of diagnosis, facilitating earlier management and genetic counseling [2,3].

In this report, we describe three rare pediatric cases of CMS with distinct genotypes and phenotypes from different families. We identified a homozygous missense variant and compound heterozygous variants in the CHRNE gene, including one novel variant and two novel compound heterozygous variants in the DPAGT1 gene associated with the limb-girdle CMS (LG-CMS) subtype. To our knowledge, only one case of this CHRNE homozygous missense variant and 11 families with LG-CMS associated with pathogenic DPAGT1 variants have been reported in the literature, making these findings exceptionally rare and reflecting the limited current understanding of this disorder [5,6,7,8,9,10,11,12]. By comparing the clinical presentation of our patients with previously reported cases, we aim to expand the current understanding of CMS and highlight the importance of early genetic testing in improving management outcomes and facilitating informed family planning.

## 2. Materials and Methods

**Patient 1**

A 4-year-old girl presented to the pediatric neurology clinic at the age of 2 years with a history of marked bilateral ptosis and fatigable muscle weakness since the age of 2 months. The parents reported that she was most active in the morning, with progressive worsening of symptoms throughout the day. Ptosis was accompanied by an inability to perform upward gaze, necessitating head tilting to watch television, which gradually progressed to head turning to see in different directions. The parents also noted that the child had weak sucking and difficulty swallowing since birth, in addition to weak cry and reduced facial expression.

Her medical history revealed no prior hospital admissions or respiratory complications. She was born to consanguineous parents after an uneventful antenatal and neonatal period. Her elder sibling was healthy. Developmental milestones were reported to be normal; however, the parents expressed concerns that her gait appeared slow and unbalanced.

**Figure 1:**
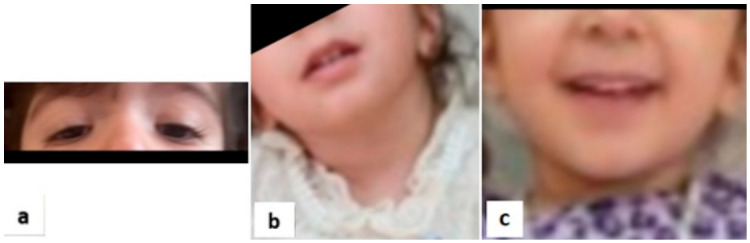
Note the bilateral ptosis (**a**), neck extension and reduced facial expressions (**b**) before treatment. Remarkable improvement in patient’s symptoms (**c**) post-pyridostigmine.

On examination, the patient had marked bilateral ptosis (Figure 1a), with a tendency to keep her neck in an extended position, in addition to diminished facial expressions (Figure 1b). Gait assessment revealed a wide-based gait; however, the patient had normal tone, power, and reflexes. Assessment of fatiguability was challenging at her age.

According to the parents, the patient’s symptoms were initially attributed to normal developmental variations, which led to a delay in recognizing the need for clinical evaluation. During the initial consultation, several noncontributory investigations were ordered, prompting the parents to seek a second opinion, which ultimately led to genetic testing and confirmation of the diagnosis. This also imposed a financial burden on the family, creating another obstacle to reaching a definitive diagnosis. Suspicion for CMS was raised following the negative serum result for AChR and MuSK antibodies. WGS revealed the presence of a homozygous variant in the CHRNE gene, c.991C>T (p.Arg331Trp). The pathogenicity of this variant has been established previously [12]. Parental genetic screening was performed to determine the carrier status of the specific point mutation. The analysis confirmed the presence of the CHRNE variant in a heterozygous state in both parents, indicating that they were carriers of this autosomal recessive disorder. The WGS testing pipeline used included mitochondrial DNA analysis and mitochondrial disorders were excluded.

Treatment with pyridostigmine was initiated at the dose of 7.5 mg twice daily and sequentially increased to the dose of 15 mg three times daily (3.5 mg/kg/day), and at follow-up, substantial improvement in ptosis, facial expressions and overall daily activity was noted (Figure 1c). Genetic counseling was provided regarding the autosomal recessive inheritance pattern and the 25% recurrence risk in future pregnancies, with preimplantation genetic testing (PGT) offered as a potential reproductive option.

**Patient 2**

**Figure 2:**
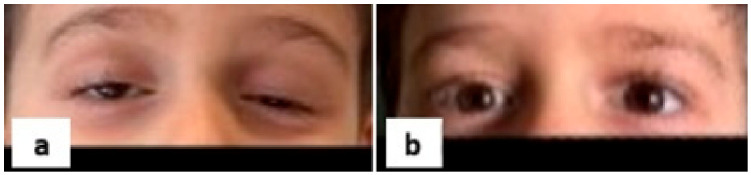
(**a**) Presence of bilateral ptosis and (**b**) improvement of ptosis post-pyridostigmine.

A 4-year-old boy presented to the pediatric neurology clinic for the evaluation of bilateral congenital ptosis and restricted eye movements. His mother reported that ptosis had been present since birth and had worsened with fatigue or sustained visual focus, such as while watching television (Figure 2a). There were no episodes of diurnal fluctuations, generalized weakness, or respiratory distress. Although the patient was less inclined to walk long distances, this was not clearly described as true fatigability.

The patient was born at term after an uneventful pregnancy and delivery. Early developmental milestones were normal. There was history of feeding difficulties, especially breastfeeding and swallowing difficulties but no hypotonia, or delayed motor milestones. The patient’s younger sibling was healthy. The parents were non-consanguineous and originated in different regions. The paternal grandmother had a history of a possible autoimmune disorder.

Upon examination, bilateral ptosis was evident and became more pronounced with fatigue or prolonged gaze. He also appeared to have some limitation in eye movements, suggestive of ophthalmoplegia, which was similarly noted during ophthalmologic evaluation. Facial and limb strength, tone, coordination, and gait were normal. He was cognitively bright and socially interactive.

Given the ocular-predominant presentation with fatigability, CMS was suspected, and congenital fibrosis of the extraocular muscles (CFEOM) was considered as the differential diagnosis. WGS revealed two heterozygous variants in the CHRNE gene: c.905C>G (p.Pro302Arg), classified as likely pathogenic, and c.1040T>C (p.Leu347Pro), classified as a variant of uncertain significance. Parental segregation analysis of the two identified CHRNE gene variants confirmed compound heterozygosity and demonstrated that the allelic variants were present in trans, confirming the molecular diagnosis of CHRNE-related CMS. No other pathogenic variants associated with neuromuscular disorders have been identified. The WGS testing pipeline used included mitochondrial DNA analysis and mitochondrial disorders were excluded. Treatment options were discussed with the family, and pyridostigmine was started at a dose of 15 mg twice daily (2 mg/kg/day). At 2-week follow-up, the parents reported a marked improvement in the child’s symptoms following treatment with pyridostigmine (Figure 2b). His neck posture improved, but he developed photophobia with severe intolerance to looking at sunlight. Photophobia is a recognized, though uncommon, cholinergic side effect of pyridostigmine. In addition, this was thought to be related to the patient not being accustomed to looking at direct sunlight with his eyes wide open. Fortunately, photophobia resolved after a few days. Approximately 2 months after the treatment, the dose of pyridostigmine needed to be increased to 15 mg three times daily, as the response appeared to decrease with the return of ptosis in the afternoon. The patient continues to be stable at the dose of 15 mg three times daily.

**Patient 3**

**Figure 3:**
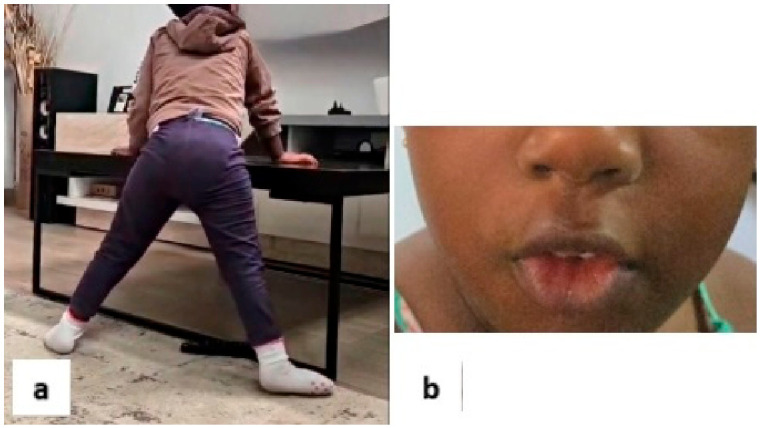
(**a**) Inability to walk without assistance and (**b**) open mouth posture.

A 3-year-old girl presented to the pediatric neurology clinic for re-evaluation of delayed motor milestones, specifically her inability to walk independently and dependence on assistance for activities of daily living (Figure 3a). Concerns first arose at three months of life due to missed milestones, particularly poor head control, leading to a diagnosis of hypotonia and the initiation of physiotherapy at four months of age. There was also a history of weak sucking and poor breastfeeding in the first two weeks of life. Her mouth was reported to be open more than 90% of the time, with associated mouth breathing.

Her medical history was significant for reduced fetal movements noted during pregnancy, which was otherwise uneventful. She was the first child to be born to non-consanguineous parents. Upon observation, the patient demonstrated an open-mouth facies (Figure 3b) and limb girdle pattern of weakness. Her behavior was described as calm.

**Figure 4:**
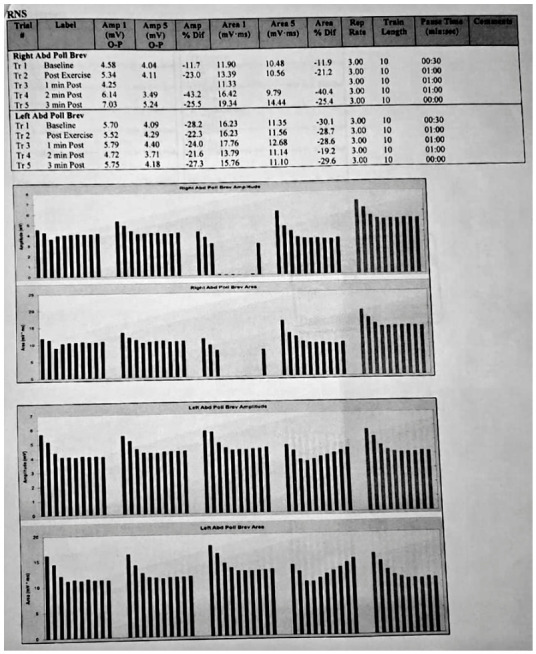
Repetitive Nerve stimulation results demonstrating decremental response.

A repetitive nerve stimulation (RNS) study performed on bilateral median nerves, recording the abductor pollicis brevis (APB) muscles, showed a decremental response with percentage amplitude change greater than 11% in all test runs. (Figure 4) WGS performed at 1 year of age revealed compound heterozygous variants c.710G>T (p.Gly237Val) and c.858C>A (p.Phe286Leu) in the DPAGT1 gene, strongly supporting a diagnosis of CMS. The WGS testing pipeline used included mitochondrial DNA analysis and mitochondrial disorders were excluded. She was initiated on oral pyridostigmine 40 mg every 4 h (Q4h) with good response. At 1-month follow-up, however, the parents reported a “wearing-off” effect, with increased floppiness and reduced energy towards the end of the 4 h dosing interval. The current dose became less effective over next 4–6 months. There is a plan to add 3,4-diaminopyridine (DAP) to determine if it can improve the symptoms.

## 3. Discussion

Mutations in 32 genes have been identified as the cause of CMS. These mutations allow CMS to be classified into subtypes according to the location of the affected proteins in the NMJ. The associated defects include eight pre-synaptic, four synaptic, fifteen postsynaptic, and five glycosylation-related defects [1]. CMS associated with severe endplate AChR deficiency arises from various homozygous or heterozygous recessive mutations in the genes encoding AChR subunits, most commonly occurring in the ε (CHRNE) subunit gene, likely because the fetal γ (CHRNG) subunit can partially compensate for the absence of the ε (CHRNE) subunit. In contrast, individuals with low-expression or null mutations in subunits other than ε (CHRNE) may not survive because no alternative subunit exists to replace their function [2].

Mutations in the DPAGT1 gene have been identified as one of the causes of glycosylation defects. DPAGT1 encodes a transmembrane enzyme responsible for N-linked protein glycosylation in the endoplasmic reticulum, a process essential for the glycosylation of AChR subunits and their export to the cell surface. Consequently, the number of AChRs is reduced [6]. According to the Human Gene Mutation Database, 145 allelic variants of CHRNE and 43 allelic variants of DPAGT1 have been reported to date [13].

Patient 1 had a homozygous missense variant in CHRNE gene: c.991C>T (p.Arg331Trp), known as R331W, which has previously been associated with the fast-channel subtype. A 2020 study reports generation of induced pluripotent stem cells from a 24-year-old female patient with CMS carrying the homozygous c.991C>T (p.Arg331Trp) mutation in CHRNE [12]. This mutation affects CHRNE protein function by slowing the rate of channel opening and accelerating the rate of ACh dissociation, thereby shortening channel burst duration [14]. Patient 2 carried a heterozygous CHRNE-gene c.1040T>C missense variant in the paternal allele and a heterozygous c.905C>G missense variant in the maternal allele. To the best of our knowledge, the first variant is novel and has not been previously reported. Thus, the patient was compound heterozygous for two CHRNE variants, c.[1040T>C] and c.[905C>G], resulting in p.(Leu347Pro) and p.(Pro302Arg). Patient 3 harbored a heterozygous DPAGT1 c.710G>T missense variant in the paternal allele and a heterozygous c.858C>A missense variant in the maternal allele. To the best of our knowledge, these DPAGT1 variants are novel and have not been previously described. Consequently, the patient was compound heterozygous for two DPAGT1 variants, c.[710G>T] and c.[858C>A], resulting in p.(Gly237Val) and p.(Phe286Leu).

The first two patients presented during early childhood with characteristic ocular features of CHRNE-related CMS, namely bilateral ptosis and ophthalmoplegia, which are among the most frequent initial findings in this subtype [1,3]. It has been reported that the extraocular and levator palpebrae muscles are disproportionately affected by their continuous low-amplitude firing pattern and higher metabolic vulnerability [1,3]. Although both our patients shared the same affected gene, their phenotypes varied considerably, reflecting the genetic and allelic heterogeneity commonly observed in CMS. Patient 1 presented with bilateral ptosis and fatigable weakness at 2 months of age, with a positive response to pyridostigmine, similar to previously reported cases of CHRNE mutations [1]. Patient 2 also presented with bilateral ptosis and partial limitation of eye movements in addition to mild speech delay, although skeletal muscle weakness was absent. The presence of a novel variant (p.Leu347Pro) may contribute to subtle phenotypic differences, supporting the idea that different allelic combinations within the same gene can produce variable clinical expression. Sex-related differences in disease severity have also been reported, with females occasionally demonstrating more severe phenotypes than males [15].

In contrast, Patient 3 presented with predominant limb muscle weakness and minimal ocular involvement. Previous studies have shown that pathogenic variants of the DPAGT1 gene cause LG-CMS associated with tubular aggregates. LG-CMS typically presents with proximal muscle weakness, although distal muscle groups may also be affected in some cases [6,7]. Craniobulbar and ocular involvement is uncommon in CMS caused by glycosylation defects, and when present, it is usually mild, manifesting as minimal ptosis or ophthalmoplegia [7,16] (Table 1).

Early genetic diagnosis is essential in patients presenting clinical features suggestive of CMS. Previous reports have noted that clinical overlap may lead to misdiagnosis, with some patients initially labeled as having autoimmune myasthenia gravis or chronic external ophthalmoplegia. In one study, the initial diagnosis of autoimmune myasthenia gravis was revised after genetic confirmation of CMS 15 years after symptom onset, preventing the continued use of inappropriate immunosuppressive therapy [3,17]. Owing to the rarity of CMS, a standardized treatment strategy has not yet been established, as only a few adequately powered treatment studies exist [1].

Reviews of pharmacological strategies have reported that acetylcholinesterase inhibitors (AChEIs), DAP, fluoxetine (FLX), and quinidine (QUIN) may be effective in some CMS patients with CHRNE mutations, although treatment responses vary depending on the specific genotype [4,16,18]. AChEIs are generally beneficial in fast-channel syndromes and primary AChR deficiencies, whereas symptoms may worsen in slow-channel syndromes [16,18]. Additionally, FLX and Quinidine may produce detrimental effects in fast-channel syndromes and in mutations involving genes, such as CHRNA1, DOK7, and MUSK [4,16,18].

Patients with DPAGT1 mutations have demonstrated a favorable response to AChEIs, although the overall effect may be mild and less pronounced in other forms of LG-CMS, such as those associated with GFPT1 variants [5,6,16]. Similar to our third patient, another study reported that the response to AChEIs may decline over time, becoming less effective as a monotherapy [7]. Beta-agonists (BA), such as salbutamol or ephedrine, have been reported as effective adjunctive therapies in CMS with CHRNE mutations, particularly in fast-channel syndromes and primary AChR deficiency [4,18,19,20]. Likewise, significant improvements have been reported in patients with DPAGT1 mutations, including those with LG-CMS, where muscle strength fluctuations were reduced and grip strength was improved [7,16,21].

### Further Discussion of the Characteristics of Variants Identified and Future Directions

Patient 1

Patient 1 had a homozygous missense variant in CHRNE gene: c.991C>T p.Arg331Trp. This variant has been demonstrated to be pathogenic through functional studies [14]. Most of the published case reports have identified this as part of compound heterozygous mutations [22]. While a 2020 study reported the generation of induced pluripotent stem cells from a 24-year-old female with CMS harboring the homozygous CHRNE c.991C>T (p.Arg331Trp) mutation, no further details regarding her clinical characteristics are available in the literature [12]. Consequently, our case potentially represents the second published instance of this variant in a homozygous state.

Patient 2

The patient was found to harbor compound heterozygous CHRNE variants in trans, including the previously reported pathogenic p.Pro302Arg variant and a rare p.Leu347Pro missense change. CHRNE c.905C>G (p.Pro302Arg) has been previously reported in congenital myasthenic syndrome in heterozygous state and shown to markedly reduce acetylcholine receptor surface expression in functional studies supporting its pathogenicity [23]. CHRNE gene: c.1040T>C (p.Leu347Pro) is currently classified as a variant of uncertain significance.

Although p.Leu347Pro is currently best regarded as a variant of uncertain significance based on available evidence, the patient’s clinical presentation was highly consistent with congenital myasthenic syndrome, and the confirmed segregation results support a recessive CHRNE-related disease mechanism. Accordingly, the overall molecular and clinical findings are strongly supportive of CHRNE-related congenital myasthenic syndrome, with p.Pro302Arg as the established pathogenic allele and p.Leu347Pro as a likely contributory second variant. Further functional studies or multiomic analysis could potentially elucidate the true impact of the uncertain variant.

Patient 3

Patient 3 was found to have compound heterozygous variants c.710G>T (p.Gly237Val) and c.858C>A (p.Phe286Leu) in the DPAGT1 gene. Both variants were classified as variants of uncertain significance (VUS) by the diagnostic laboratory. However, several lines of evidence support their likely pathogenic role as per the ACMG (American College of Medical Genetics and Genomics) standards [24]. First, both variants are rare or absent in population databases like GNOMAD, supporting a moderate level of evidence for pathogenicity (PM2) [25]. Second, the identification of these variants in a compound heterozygous state is consistent with the known autosomal recessive inheritance pattern of DPAGT1-related CMS and provides additional support (PM3), as they were found to be in trans. Third, computational prediction using AlphaMissense suggests a deleterious effect on protein function for both variants [26]. While in silico tools should not be used in isolation, they provide supporting evidence (PP3) when interpreted in the context of other data.

Critically, the patient’s phenotype demonstrates a high degree of specificity for DPAGT1-related CMS, fulfilling supporting clinical evidence (PP4). The presentation of early-onset fatigable weakness with a limb-girdle distribution, combined with electrophysiological evidence of a neuromuscular transmission defect, aligns closely with previously reported cases. This genotype–phenotype concordance is a key factor in variant interpretation, particularly in rare disorders where functional validation is often unavailable.

Despite these supportive findings, we acknowledge the key limitations in assigning pathogenicity to these variants. Functional studies demonstrating the impact of these specific variants on DPAGT1 enzymatic activity or AChR glycosylation were not performed, and therefore strong functional evidence (PS3) is lacking. Additionally, these variants have not been previously reported in affected individuals, precluding the use of case enrichment data.

## 4. Conclusions

In conclusion, this report highlights the importance of early genetic testing for pediatric patients with phenotypes suggestive of CMS, given the considerable clinical overlap with other neuromuscular disorders. Early and accurate diagnosis is essential to prevent misdiagnosis and the use of potentially harmful therapies that may be contraindicated for specific genetic subtypes. In addition, molecular confirmation supports neurologists in providing appropriate genetic counseling for affected families, including guidance on reproductive options, such as in vitro fertilization (IVF) with PGT to prevent disease transmission in future pregnancies. Furthermore, our findings expand the current CMS mutational spectrum, as we identified a second case of the homozygous R311W mutation and a novel compound heterozygous variant c.[1040T>C]; [905C>G] in the CHRNE gene, as well as two novel compound heterozygous variants c.[710G>T]; [858C>A] in the DPAGT1 gene.

## Figures and Tables

**Table 1:** Characteristic comparison of the patients.

	Case 1	Case 2	Case 3
Sex	Female	Male	Female
Gene symbol	CHRNE	CHRNE	DPAGT1
Descriptive name	Primary AChR deficiency	Primary AChR deficiency	Defect of glycosylation
Genetic mutation type	Homozygous	Compound heterozygous	Compound heterozygous
Age at presentation	2 years	4 years	3 years
Symptom onset	2 months	Since birth	2 weeks
Ocular symptoms	Bilateral ptosis and limited upward gaze	Bilateral ptosis and restricted eye movements	None
Bulbar involvement	Facial weakness and weak sucking	None	Facial weakness and weak sucking
Limb-girdle weakness	Mild	None	Severe
Motor developmental delay	None	None	Present
Treatment/Response	Pyridostigmine/Effective	Pyridostigmine/Effective	Pyridostigmine and its declining effectiveness

## Data Availability

Data supporting the findings of this study are not publicly available due to patient confidentiality and privacy considerations. Additional data may be made available from the corresponding author upon request and subject to institutional ethical approval.

## Abbreviations

The following abbreviations are used in this manuscript:

CMS	Congenital myasthenic syndrome
NMJ	Neuromuscular junction
EMG	Electromyography
AChR	Acetylcholine receptor
MuSK	Muscle-specific kinase
WGS	Whole genome sequencing
LG-CMS	Limb-girdle congenital myasthenic syndrome
CFEOM	Congenital fibrosis of extraocular muscles
RNS	Repetitive nerve stimulation
APB	Abductor pollicis brevis
Q4h	Every 4 h
DAP	3,4-diaminopyridine
AChEIs	Acetylcholine esterase inhibitors
FLX	Fluoxetine
QUIN	Quinidine
BA	Beta-agonists
IVF	In vitro fertilization
PGT	Preimplantation genetic testing
PM2	Pathogenic, Moderate #2 Moderate evidence: Absent from controls (or ultra-rare ≤ 0.0001 in gnomAD/ExAC).
PM3	Pathogenic, Moderate #3 Moderate evidence: Detected in trans with a pathogenic variant (recessive disorders)
PP3	Pathogenic, Poorly established #3 Supporting evidence: Multiple computational tools predict a deleterious effect
PP4	Pathogenic, Poorly established #4 Supporting evidence: Patient’s phenotype highly specific for a disorder with a single genetic etiology
PS3	Pathogenic, Strong #3 Strong evidence: Well-established functional studies supportive of damaging effect
VUS	Variant of Uncertain Significance Classification when evidence is insufficient to call pathogenic or benign
